# Novel Resorbable and Osteoconductive Calcium Silicophosphate Scaffold Induced Bone Formation

**DOI:** 10.3390/ma9090785

**Published:** 2016-09-20

**Authors:** Patricia Ros-Tárraga, Patricia Mazón, Miguel A. Rodríguez, Luis Meseguer-Olmo, Piedad N. De Aza

**Affiliations:** 1 Grupo de Investigación en Regeneración y Reparación de Tejidos, UCAM—Universidad Católica San Antonio de Murcia, Guadalupe, Murcia 30107, Spain; p.ros.tarraga@gmail.com; 2 Departamento de Materiales, Óptica y Tecnologia Electrónica, Universidad Miguel Hernández, Avda. Universidad s/n, Elche, Alicante 03202, Spain; pmazon@umh.es; 3 Instituto de Cerámica y Vidrio, ICV-CSIC, C/Kelsen 5, Madrid 28049, Spain; mar@icv.csic.es; 4 Service of Orthopaedic at Arrixaca University Hospital, UCAM-Catholic University of Murcia, Murcia 30120, Spain; lmeseguer.doc@gmail.com; 5 Instituto de Bioingenieria, Universidad Miguel Hernandez, Avda. Ferrocarril s/n, Elche, Alicante 03202, Spain

**Keywords:** porous bioceramic, calcium silicophosphate, in vivo response, biocompatibility, histology, scanning electron microscopy

## Abstract

This aim of this research was to develop a novel ceramic scaffold to evaluate the response of bone after ceramic implantation in New Zealand (NZ) rabbits. Ceramics were prepared by the polymer replication method and inserted into NZ rabbits. Macroporous scaffolds with interconnected round-shaped pores (0.5–1.5 mm = were prepared). The scaffold acted as a physical support where cells with osteoblastic capability were found to migrate, develop processes, and newly immature and mature bone tissue colonized on the surface (initially) and in the material’s interior. The new ceramic induced about 62.18% ± 2.28% of new bone and almost complete degradation after six healing months. An elemental analysis showed that the gradual diffusion of Ca and Si ions from scaffolds into newly formed bone formed part of the biomaterial’s resorption process. Histological and radiological studies demonstrated that this porous ceramic scaffold showed biocompatibility and excellent osteointegration and osteoinductive capacity, with no interposition of fibrous tissue between the implanted material and the hematopoietic bone marrow interphase, nor any immune response after six months of implantation. No histological changes were observed in the various organs studied (para-aortic lymph nodes, liver, kidney and lung) as a result of degradation products being released.

## 1. Introduction

The growing number of bone reconstructive surgery types, along with progressively prolonged life expectancy in developed countries, make research and the development of alternatives to natural bone grafts increasingly important. Bioactive scaffolds play an important role in tissue engineering [1,2].

A bone substitute material should display ‘bimodal’ behavior, which, in early differentiation stages, allows osteoblasts to build bridges between different sizes of grain and to integrate with other osteoblasts to support both proliferation and differentiation. New bone formation has been stimulated by the activation of mesenchymal stem cells and their absorption onto surfaces with nanoscale topographic features [3]. The ultimate goal is to unite fully differentiated osteoblasts that support bone matrix production. This requires a porous structure with nanopores, micropores and macropores, all of which are involved in different stages of absorption, adhesion and bone material deposition on and within the bone substitute material [4]. 

How to effectively improve the attachment of cells in the interior of large-sized scaffolds is still a significant bone tissue engineering challenge. Some studies have suggested that macropores with a pore diameter that falls within the 50–300 μm range are beneficial for cell attachment, proliferation and vascularization, while the micropores within the 0.5–10 μm range are desired to provide the effective delivery of nutrients and physical cues to enhance cell response. It now seems acceptable that a minimum interconnect size of ~100 μm is needed for mineralized tissue ingrowth [1,3,4]. These results underline the need for developing new technologies to produce strong scaffolds with controlled porosity [5,6]. Part of this work focuses on the sintering of porous calcium silicophosphate scaffolds with macro- and microporosity by the polymer replication method.

Regarding silicon that contains calcium phosphate materials, the effect of Si on healthy bone and connective tissues is well-known. Silicon modifies the material properties and improves the biological activity of silicon that contains CaP materials. Thus, they have been widely studied as biomaterial for osseous repair [7,8].

The interest in rich-silico phosphate biomaterial is currently increasing because of its good bioactivity response and low cytotoxicity [9,10]. 

In this context, the compositions that belong to subsystem Nurse’s A-phase- silcocarnotite within system Ca_3_(PO_4_)_2_–Ca_2_SiO_4_ are promising candidates for preparing new ceramic bone implants [11]. Nurse’s A-phase is a solid solution with an approximate composition of 7CaOP_2_O_5_2SiO_2_ [12,13], which should not be confused with the mineral of the same composition identified by Nagelshmidt in 1937 [14]. Silicocarnotite (5CaOP_2_O_5_SiO_2_) can be defined as calcium silicophosphate with a carnotite structure [7,15].

The main purpose of the present study was to investigate the effect of pore morphology on its in vivo osteoconductivity and resorption process of new calcium silicophosphate ceramic scaffolds obtained by the polymer replication method.

## 2. Results

### 2.1. Scaffold Characterization

**Figure 1:**
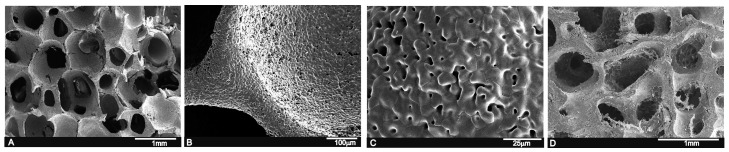
(**A**) SEM view of the calcium silicophosphate scaffolds obtained by the polymer replication method at low magnification, which reveals high porosity and interconnectivity; (**B**,**C**) the high magnification view of the scaffold indicates largely distributed microporosity; and (**D**) human cancellous bone tissue for comparison purposes.

By the polymer replication method, it was possible to produce highly porous calcium silicophosphate scaffolds with a striking similarity to human cancellous bone tissue (see Figure 1A,D). The SEM observations revealed pore diameters that ranged from 200 μm to 1.0 mm, and a pore wall thickness of ~50 μm (Figure 1B). Micropores from 1 μm to 10 μm were also viewed on pore walls and struts (Figure 1C). The scaffold composition, determined by a quantitative analysis by EDS at different sample points, was around 15.1 wt % SiO_2_, 58.2 wt % CaO and 26.6 wt % P_2_O_5_.

Apparent density was 75 gr·cm^−3^, which meant total porosity was 76%. Hg porosimetry (Poremaster-60GT, Quantachrome, Boyton Beach, FL, USA) was carried out. It indicated that 15% of pores were bigger than 1 mm, 20% ranged between 1000 and 100 μm, the rest were below 100 μm, and this distribution was centered around 10 μm. The material’s mechanical strength was sufficient for handling and placing it inside the surgical site.

### 2.2. Radiological Study

**Figure 2:**
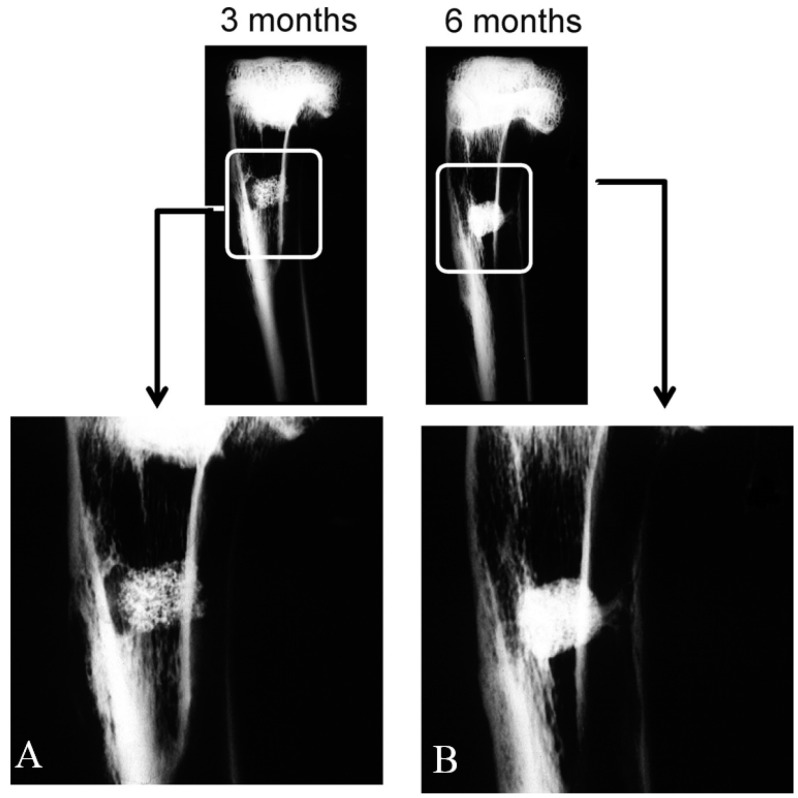
Antero-posterior X-rays of the bone section that contained the implants after three (**A**) and six months (**B**) post-implantation.

Radiological density varied for each implantation time. X-ray plains revealed that the scaffold (Figure 2A) gave a rectangular image of great radiopacity with an irregular mottled look and a smooth outline at three months post-implantation. This aspect facilitated the identification of the trabecular bone implant in it. The scaffold showed correct integration and partial resorption.

Six months after the implantation period (Figure 2B), the material exhibited an irregular rectangular morphology and uneven outlines. It was denser at the material-bone marrow zones interface than the adjacent cancellous bone as a result of new mature bone formation in pores, which correlated with the histological findings. No alterations, loss of their normal distribution pattern in the adjacent bone trabeculae or ectopic bone formation was/observed.

### 2.3. Histological Evaluation

**Figure 3:**
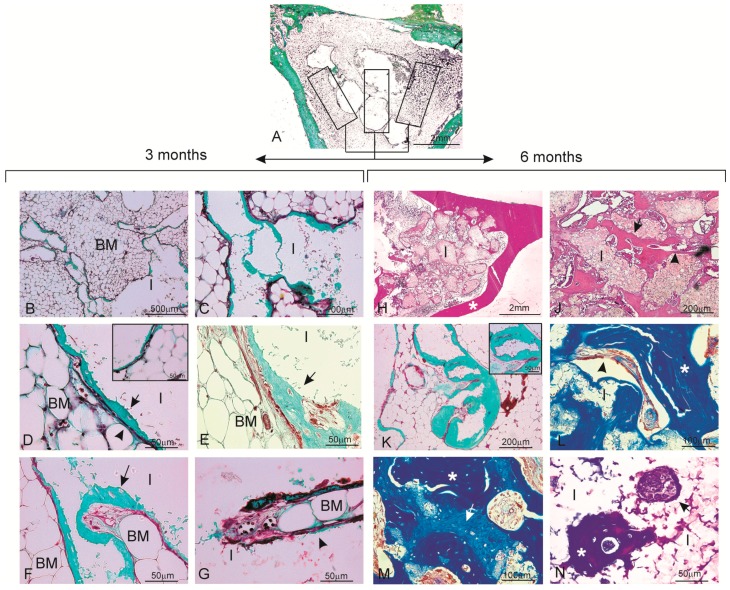
Histological analysis of the material samples stained with Masson’s Trichome (**A**–**G**,**K**–**N**) and hematoxylin-eosin (**H**,**J**) three months (**A**–**G**) and six months (**H**–**N**) after implantation. (**A**) panoramic image of the implant section together with the different areas drawn for the descriptive histological study as being representative of the histological study at three and six months. The ceramic implant, which was removed during histological processing, is seen as a blank/empty space; (**B**,**C**) the whole material is observed in close relation with the hematopoietic bone marrow-interior of the pores colonized by hematopoietic bone marrow elements (BM: bone marrow; I: implant); (**D**) blood capillaries located between a thin osteoid tissue layer and surrounding bone marrow material. The insert of the figure shows some areas of the new osteoid tissue with osteocytic lacunae (osteoblast) and with osteocytes inside (BM: bone marrow; I: implant, arrow head: capillary blood; arrow: osteoid); (**E**) image corresponding to the central area of the material with newly formed bone tissue with the trabecular bone characteristic (arrow); (**F,G**) new bone colonization of the implanted material’s interior (arrow); (**H**) after six months of implantation, the implanted material is completely enveloped by new bone tissue and penetrates large areas (* cortical bone); (**J**) Presence of bone tissue in both the periphery and interior of the implanted material; (**K**) bone tissue deposition occurred mainly in the form of layers. The insert shows a pore filled with new bone formation; (**L**) bone with normal mature characteristics and in-growth inside one of the implanted material’s pores; (**M**) differences in the **blue** staining shade reveals the presence of scattered areas of older bone tissue (**light blue**) (arrow) and newly formed bone (**dark blue**) (*); and (**N**) presence of two nodular structures within the material. Cuboidal osteoblastic cells are seen to line the surface of the developing osteoid tissue (arrow) (BM: bone marrow; I: implant).

The histological sections of the materials were analyzed at three months (Figure 3A–G) and six months post-implantation (Figure 3H–N). An analysis of the samples stained with Masson’s Trichome and hematoxylin-eosin confirmed the observations made by radiography. All the animals survived the three- and six-month study periods and presented no evidence for inflammation or infection at the implantation sites. Figure 3A shows a panoramic image of the cylinders implanted across the rabbit proximal in the tibia metaphysis at three months post-implantation as being representative of the histological study at three and six months. Rectangles were traced manually to create an individual region of interest (ROI) in the periphery and center of the material. The descriptive histological observations of bone tissue responses are summarized in Table 1.

#### 2.3.1. Three Months

Details of the implant haematopoietic bone marrow interface can be seen at the implant site in Figure 3B. While processing samples, the material was occasionally dragged and left some empty spaces in the histological sections. At a higher magnification (Figure 3C), the colonization process of the scaffold started in the periphery and then penetrated throughout implant porosity.

It is important to highlight the absence of either inflammatory cells or fibrous connective tissue formation in the vicinity of the implanted material and around the newly formed woven bone, which would otherwise imply bone tissue intolerance to the implant (Figure 3D). This fact allowed the arrival of the blood capillaries (arrow head) located between a thin layer of osteoid tissue (arrow) and bone marrow to the surrounding material. Osteoid tissue was formed and detected as a thin layer located directly on the material surface at the implantation site (arrow head). In some osteoid tissue areas, osteocytes lacunae (osteoplast), with osteocytes inside, were viewed (Figure 3D—inset). Presence of poorly inflammatory cells (macrophages and lymphocytes) was observed on the material’s edges. No multinucleated giant cells were observed during this period. In other areas, newly formed bone tissue took the trabecular bone characteristic (arrow) in connection with the blood capillaries that delimited bone marrow. These findings can be observed in both the peripheral portions and the material’s interior at this time point (Figure 3E). 

**Figure 4:**
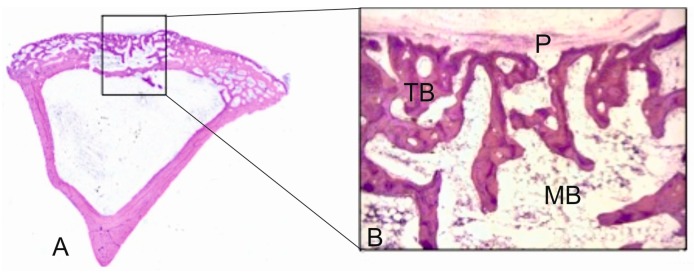
(**A**) Panoramic image of the control sample after three months of healing (31.2×); (**B**) representative histological microphotograph showing the bone defect partially filled with the newly formed trabecular bone (TB), hematopoietic bone marrow (BM) and periosteum layer (P) on the surface (125×).

The presence of thin layers of osteoid with osteoblasts were observed in the implanted material, along with elements of the bone marrow and vascular capillary that invaded the scaffold (Figure 3F). Practically none of the macrophages were observed in the evaluated sections. The fast colonization of the implanted material’s interior was also noteworthy. At a higher magnification, Figure 3G shows how a vascular axis penetrated the material, and also the beginning of the bone formation process (arrow head). In the ungrafted specimens (controls), the cortical defect was repaired by newly formed trabecular bone tissue with normal histological characteristics (Figure 4). The increased presence of osseous trabecula was located on the surface that came in contact next to the periosteum layer. No alterations in the bone marrow that occupied the intertrabecular spaces were observed.

#### 2.3.2. Six Months

The most relevant finding during this study period was that the implanted material was completely enveloped by the new bone tissue, which penetrated large areas and mainly replaced the implanted material. Likewise, the continuity of the newly formed bone tissue came into close contact with the overlying cortical bone (*) (Figure 3H). The material’s pores increased in size as a result of degradation, which led to a more irregular surface and favored bone in-growth.

At a higher magnification, Figure 3J highlights the presence of bone tissue on the periphery and in the implanted material’s interior. Neither the immune response nor the formation of interposed fibrous structures at the material-bone tissue bone interface was observed (arrow). In the central implant area (Figure 3K), the new bone tissue deposition occurred mainly in the form of layers with a cortical architecture, and numerous osteocytes lacunae were noted. Figure 3L shows the vast quantity of mature bone tissue (*), and also new bone growing trabecularly adjacent to the material and inside the pores that come into close contact with the partially reabsorbed material (creeping substitution).

Newly formed bone tissue was observed at various maturation levels (Figure 3M). Mature bone with an osteon regular structure was surrounded by the areas where the new bony tissue was still in its early maturity stages (osteoid). Differences in the blue staining shade revealed scattered areas of older bone tissue (dark blue, *) and newly formed bone (light blue, arrow). During this period, the bone in all the scaffolds predominately comprised highly cellular disorganized woven and mature bone. Bone surfaces were populated by highly active cuboidal osteoblastic cells, which is consistent with mesenchymal (membranous) type bone ossification. Furthermore, presence of cement lines and filled osteocytic lacunae demonstrated healthy new mature bone apposition. 

Finally, Figure 3N corresponds to the scaffold’s interior. Presence of two nodular structures was evidenced, and one was composed of cells of a macrophage aspect and bone tissue that partially coated it. The other node dominated the presence of islets with osteoid and osteoblastic borders, and acquired the appearance of trabecular bone and newly formed trabecular bone. However, it was noteworthy that there was no inflammatory response and that fibrous tissue surrounded the material.

**Figure 5:**
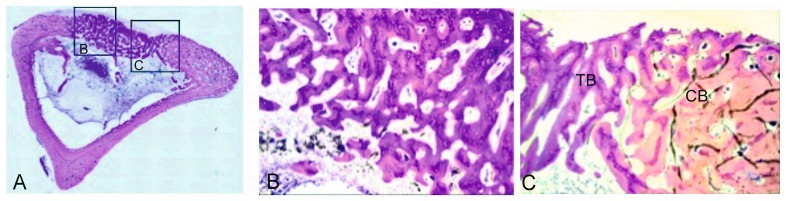
Representative histological microphotographs of the control samples after six months. (**A**) panoramic image (31.2×); (**B**) detail of the new trabecular bone (TB) (125×); and (**C**) detail of the boundary between compact bone (CB) and the newly formed trabecular bone (200×).

During the same period, the control samples showed how the bone defect was completely occupied by newly formed lamellar bone (Figure 5A). The new trabecular bone was thicker and composed of irregular lamellae with active osteocytes (Figure 5B,C). These histological findings formed part of the bone remodeling process in the endochondral ossification context.

### 2.4. Histomorphometric Analysis

Histomorphometric analyses were carried out to establish the BIC values for the material, and gave high BIC values (67.30% ± 1.41 *) (with close contact observed). New bone ingrowth, defect closure and residual biomaterial were recorded and analyzed (Table 2). In the control samples, the newly formed bone in the cortical defect increased, and even in a smaller amount than in the grafted defects. 

### 2.5. Scanning Electron Microscopy Findings

**Figure 6:**
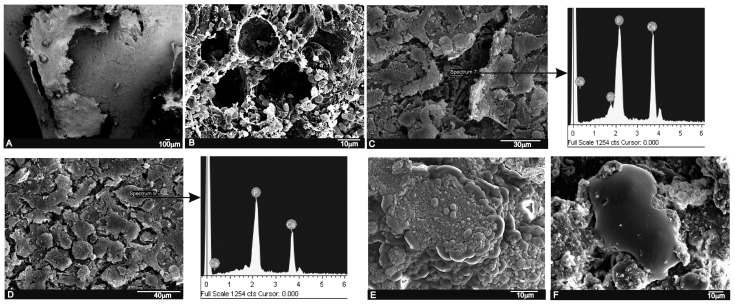
SEM images and EDS analyses of the scaffold implant after three months (**A**–**C**) and six months (**D**–**F**) of implantation. (**A**) general panoramic section of the tibia that contained the implant; (**B**) an abundant blood-fibrin structure partially lined the material’s pores; (**C**) implant surface partially covered by an immature bone tissue and the EDS analysis; (**D**) the implant surface covered by newly formed bone tissue and the EDS analysis; (**E**) detail of the immature bone tissue’s globular aspect; and (**F**) a macrophagic cell in close contact with the granular deposit from the implanted material.

A strong correlation was found between the SEM implantation results and the relevant histological findings. A low magnification cross-section at the implantation level revealed the tibia that contained the implant (Figure 6A). 

In the SEM images, particles of implants were seen to be white-gray due to a low organic content and a relatively high Ca/P/Si ratio, whereas the newly formed bone was darker gray given the presence of collagen, bone marrow and fat. After three months of implantation, the scaffold material’s general architecture was still visible. There were various elements from the hematopoietic bone marrow (erythrocytes, lymphocytes, platelets, macrophages) embedded in a fibrillar network, which surrounded the surface and were inside the material’s pores (Figure 6B). The formation of a thin immature bone tissue layer, which partially covered the implant, was observed directly on the implant surface (Figure 6C). The EDS analysis of the underlayer was composed of a Ca-P-Si compound. 

After six months of implantation, the whole ceramic implant surface was covered by a newly formed bone tissue (Figure 6D). The new bone layer was composed of Ca-P, mainly with traces of Si due to the gradual diffusion of Si ions from the scaffolds into the newly forming bone, which formed part of the biomaterial’s resorption process. Details of the new bone layer show the characteristic globular pattern of a carbonate hydroxyapatite (Figure 6E). In some areas, another distinguishing characteristic was found: presence of irregularly shaped voluminous cells with cytoplasmatic extensions, which appeared macrophagic and were in close contact with small accumulations of the implanted particle which they surrounded, and different resorption process stages were seen.

**Figure 7:**
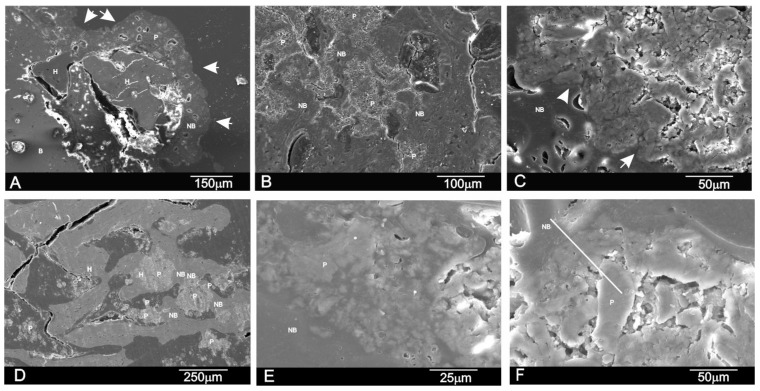
SEM images of the cross-section of the scaffold after (**A**–**C**) three months and (**D**–**F**) six months of implantation. (H indicates a pore filled with new bone, P refers to implant particles as a result of the degradation process, while NB refers to new bone tissue. Arrows indicate an irregular new bone boundary with partially degraded implant particles inside).

Figure 7 shows the SEM image of the polished cross-section of the implant at three and six months. The cross-sectional SEM evaluation confirmed that the residual scaffold particles were surrounded by the newly formed bone, which presented mature bone characteristics with well-organized lamellae and numerous small osteocytic lacunae (Figure 7A,B,D). The bone-to-biomaterial interface was characterized by small numbers of newly formed bone projections, which reached scaffold particles (Figure 7C,E). The new bone that filled pores and particles was partially embedded in the new bone tissue (Figure 7A,B,D). Bony integration was well advanced in all the samples, and bone penetration was complete throughout deep and central zones.

Figure 7F shows a representative SEM cross-section where the EDS analysis was done. An analysis was carried out at various points, following the recommendations of Lindgren et al. 2010 to take different points of interest from the middle and periphery of samples to detect changes in the Si/Ca/P ratios. Table 3 provides the descriptive statistics for our database. We found that active biomaterials resorption was underway. The EDS analysis of the residual scaffold particles in the retrieved tissue revealed a Ca/P ratio of varying relative proportions. An elemental analysis of the residual scaffold at different points revealed some categories of particles with different mean Ca/P ratios according to degradation status. For the statistical data, an elemental analysis indicated a relatively high Ca/P ratio in the residual scaffolds (2.62 < Ca/P < 2.68) and at the bone interface (2.20 < Ca/P < 2.36) compared to the new bone (1.89 < Ca/P < 1.97). The specific Si ion concentration in the scaffold lowered from 10.77 ± 0.03 wt % in the material before being implanted to 6.89 < Si < 7.46 after implantation and at the bone interface (0.26 < Si < 0.12) compared to the new bone (0.07 < Si < 0.04). This finding suggests that the gradual diffusion of the Ca and Si ions from the biomaterial to the newly forming bone at the interface forms part of the biomaterial’s resorption process.

## 3. Discussion

Interconnected Si-Ca-P porous scaffolds, where a porous polymer sponge was used as a template, were processed to be used as novel material with osteoconductive properties for bone reconstruction, with similar properties to autologous bone grafts. The main findings showed that the porous scaffold degraded over the experimental set points and allowed new bone tissue formation.

The porous bioactive scaffold also induced new bone tissue formation inside the material in three ways: (i) invasion of newly formed bone tissue on the material’s surface using the network of interconnected pores, with the material starting from the periphery toward its center; (ii) macrophage activity that precedes invasion or penetration of bone marrow, which provides a capillary axis accompanied with vascular cells, and an osteoblastic line; and (iii) the material per se is able to create the microenvironment required around it to locally carry out the osteogenic differentiation of the osteogenic precursor cells contained in the hematopoietic bone marrow.

Porous bioactive scaffolds are most interesting to be used as bone substitutes in the bone tissue engineering field [16]. High bioactivity and adequate scaffold porosity are essential characteristics to stimulate osteoprogenitor cells and to support bone in-growth. Furthermore, resorption of the material with the same bone formation rate is required [17]. Several in vivo studies have demonstrated that Si influences bone mineralization [9,10], metabolism [18,19], collagen synthesis [20,21] and crosslinking [22]. These findings fall in line with the results of the current study, which revealed continuous newly bone tissue in-growth in the defect area, inside pores and in the spaces left by the degraded scaffold. After implantation, the dissolution of Si and Ca ions from the scaffold to bone tissue stimulates the formation of a carbonate-hydroxyapatite layer, which acts as a template for osteoblast growth and can affect osteogenesis [3,4,23,24]. High porosity and adequate pore sizes are essential factors for effective bone substitute material. Scaffolds with an optimal pore size allow bone in-growth and support neovascularization. Both the pore size and porosity of the bioactive scaffold used herein indicate its morphological characteristics, which make it suitable for being used as a bone graft. Moreover, the histological findings demonstrated that the scaffold degraded over time and that degradation happened according to the tissue in-growth rate. Besides adequate porosity, proper scaffold degradation is also essential for the process to happen since new bone tissue formation needs space to grow in [3,4,23,24].

Several techniques have been used to assess the biomaterial-to-bone tissue interface. Many evaluations of the interface using LM, SEM, and TEM have shown how the same structure is represented differently depending on the examination method selected [25,26]. The traditional method used during bone regeneration, histological staining followed by examination under a light microscope, provides substantial information. However, its low spatial resolution does not provide ultrastructural information. The precision and reliability of a histomorphometric study of newly formed bone depends on the correct identification and ultrastructural characterization of all the cellular components that could play a role in the osseointegration process. LM lacks the resolving power required for detailed structural analyses. Electron microscopy techniques can help characterize the morphological changes that occur during osseointegration. Despite certain limitations [27], transmission electron microscopy has been successfully used to describe the cellular components of newly formed bone.

SEM, used to examine bone-to-biomaterial interfaces, was first reported by Jasty et al. [28]. This method has been employed mainly for descriptive studies performed with calcified tissue [29,30]. SEM images can be used to highlight contrasts between areas of different chemical compositions, and this technique is especially effective whenever average atomic numbers of the components of each region vary. Since SEM signal intensity depends on the sample’s mean atomic number, the SEM technique not only serves to distinguish inorganic features, but also offers the interesting possibility of identifying ultrastructural cell components and their micromorphological details.

In the present study, the LM, SEM and EDS analyses revealed a close relation between the newly formed bone matrix and the scaffold particle surface. The elemental analysis of bone tissue demonstrated the presence of calcium and phosphorus, which indicated the presence of mineralized bone tissue on the particle surface (Table 2 and Table 3). This observation suggests that the scaffold surface could provide an optimal stratum for bone tissue in-growth. The SEM analysis also showed that the new bone matrix had grown over the scaffold surface, and had completely penetrated the deep central zones through its porous structure. After six months of implantation, the scaffold implant had well integrated into the host tissue, and had formed an irregular surface boundary caused by the material’s gradual degradation (Figure 6 and Figure 7). The interface developed between the implant and the surrounded tissue was characterized by the intermittent presence of the calcium phosphate phase, which corresponded to new bone tissue in structure and morphology terms.

The new bone ingrowths in the implant were more evident at six months and advanced into the spaces between the exposed scaffold particles in the implant to form a characteristic interlocking pattern at the interface as the process moved further into the implant. SEM (Figure 7) showed a massive bone colonization of the implant through the original scaffold pores, caused by the structure’s gradual dissolution. Due to these advanced processes, the scaffold material’s free particles were found in many areas across the restructuring implant. Densities at the bone-ceramic interface and inside the material gradually and significantly reduced. This indicates that the resortive process went from the periphery to the center, and it initiated in an early material implantation stage by a cellular mechanism (macrophage cells). At the end of the study (six months), the central part of the implanted material remained partially degraded as it was in the histological and radiological images (Figure 2 and Figure 3). However, a lot of new bone was observed in bone defects treated with the scaffold, in which 62.18% ± 2.28% of the bone defect was filled by the newly formed bone after six months of implantation (Table 2). 

We conclude that the results of this initial research confirmed our hypothesis that the high resorbable porous bioactive calcium silicophosphate scaffold has an adequate porosity structure and is able to support bone tissue in-growth by new bone, while gradually being resorbed by the cell-mediated process at the same time. Thus, it constitutes a promising alternative to be used as bone grafts for tissue engineering. Future research should be conducted using other scaffolds made from standard biomaterials, such as Si-HA or Si-TCP. The ceramic’s biological performance should be investigated in different bone defect models and animals, and probably with long-term assays, as proposed in International Standard ISO-10993-5.

## 4. Materials and Methods

### 4.1. Biomaterial

In this study porous scaffolds, which corresponded to the 28.39 wt % Nurse’A—71.61 wt % Silicocarnotite composition, were produced by the polymer replication method. 

Nurse’s A (7CaOP2O52SiO2) and Silicocarnotite (5CaOP2O5SiO2) ceramic powders synthesized previously in our laboratory were used as starting materials. Details of the technique and the characterization of the starting materials can be found in previous publications [7,8,13]. First, the desired proportions of each component were weighed on an analytical balance and powder was thoroughly attrition-milled in alcoholic media (isopropilic alcohol) using ZrO_2_-Y_2_O_3_ balls (1 mm diameter) for 4 h. The resulting particle size was 2.1 μm measured with laser scattering particle size equipment (Mastersizer, Malvern, UK). Ceramic slurry was prepared with 60% solid contents in water media. Dolapix CE-64 (Zschimmer Schwartz, Lahnstein, Germany) was added as a defloculant (1 wt %) and Optapix PAF-35 (Zschimmer Schwartz, Lahnstein, Germany) as a binder (3 wt %). The powder:water ratio was 60:40.

Scaffolds were prepared using polyurethane sponges with open cells (60 ppi) as a template. Sponges were impregnated with ceramic slurry and sintered at 1450 °C for 2 h with 5 °C/min as the heating and cooling rates. Then, powder was turned off and samples were allowed to cool inside the furnace for 24 h. Next cylindrical scaffolds (5 ± 1 mm diameter and 6 ± 0.3 mm long) were cleaned and washed several times in sterile PBS solution, dried at 37 °C, and finally sterilized by gas plasma (Sterrad-100S, Irvine, CA, USA) and kept in individual packages under sterile conditions until implantation.

The microstructure of the scaffolds was characterized by scanning electron microscopy (SEM-Hitachi S-3500N, Ibaraki, Japan). The chemical composition was qualitatively determined by an Energy Dispersive X-ray Spectroscopy system (EDS-INCA system, by Oxford Instruments Analytical, High Wycombe, UK).

### 4.2. Animals and Surgical Procedure

Mature male New Zealand (NZ) rabbits (*n* = 8, 4.0 ± 0.3 kg) were used. Animals were divided and randomly assigned (simple randomization) into two time groups of four animals (*n*1 = 4, *n*2 = 4) according to the previously set 3- and 6-month periods. Animals were not placed on a special diet, but received feed and water ad libitum. Animals were housed in individual standard steel cages and maintained in a 12:12 h light dark cycle under controlled environmental conditions. All the animals were allowed 1 week from their arrival to facilitate acclimation.

The study protocol was examined and approved by the Institutional Ethic and Animal Experimentation Committee of the Miguel Hernandez University according to Spanish Government Guidelines and European Community Guidelines for animal care (authorized no. 2014/VSC/PEA/00056 tipo2).

Animals were pre-medicated and anesthetized by an intramuscular (im) injection of Atropine sulfate, 0.3 mg·kg^−1^ and hydrochloride of clorpromazine, 10 mg·kg^−1^, and then hydrochlorate of ketamine, 50 mg·kg^−1^ and xylazine 5 mg·kg^−1^. As an antibiotic prophylaxis, a single dose of Enrofloxacin 2.5 mg·kg^−1^ im (Virbac, Barcelona, Spain) was provided. Both legs were shaved and washed with Chlorhexidine^®^ (Bohm SA, Madrid, Spain). Then Betadine^®^ (Meda Manufacturing, Bourdeaux, France) was applied and the surgical area was covered with a sterile drape. Afterward, a longitudinal incision (1.2–1.5 cm long) was made along the medial aspect in the proximal metaphysial area of each tibia. Subcutaneous tissue, fascia and periostium were dissected to expose the medial surface of the tibia. An end-cutting bur (5 mm diameter) was connected to a micromotor at low revolutions with continuous saline irrigation/suction to avoid overheating and thermal damage to bone, and was used to create an unicortical bone defect (5 mm diameter) to avoid invading the medullary cavity. Sufficient hemostasis was achieved. Then, the defect was thoroughly washed several times with physiological saline before performing implantation and removing bone debris to avoid it entering the defect. Porous cylindrical implants were press-fit placed into the defects to ensure initial stability. Identical osseous defect at the contralateral tibia remained empty (ungrafted) as a control. 

Afterward, wounds were carefully and closed sutured by a meticulous technique (anatomical layers) with continuous absorbable sutures (Vicryl™ 3/0 (Agatho AB, Lidingo, Sweden) for deep planes and Vicryl Rapid™ 3/0 for skin). Then, a single local anesthetic (0.2 mL·kg^−1^, chlorhydrate lidocaine 2%, subcutaneously) was provided routinely at the surgery site to avoid immediate postoperative pain. On the first three post-surgery days, animals were given a subcutaneous injection of 0.1 mL·kg^−1^ of Tolphenamic acide twice a day (every 12 h) as an analgesic control. They were allowed to freely move (limb loading) immediately after restoring from anesthesia. They all survived the 3- and 6-month study periods, and surgical wounds healed with no complications or infection. Therefore, all the animals were included in the study. Total limb loading was allowed after restoring from anesthesia.

At the end of the 3- and 6-month periods, animals were euthanized under sedation (hydrochlorate of ketamine, 50 mg·kg^−1^ im), with an overdose (0.5 mL) of pentobarbital sodium (Dolethal™, Lab. Vetoquinal, Cedex, France) intracardiacally. To remove the implanted area, the same surgical procedure as that described above was followed.

### 4.3. Radiographic Imaging

Two X-ray images (standard antero-posterior and lateral projection angles) were obtained of the area of the bone-containing implants, which was cleaned of adherent soft tissue by the Kodak RVG 6100 Digital Radiography System (Kodak DS, Rochester, NY, USA) with an X-ray taken at 32 kV, 40 mA by automatic light metering. Radiographs of all the specimens were taken. Images were used to observe changes in the morphology and radiological density (radiopacity) descriptive levels of the material in the medullar and cortical areas where defects were created.

### 4.4. Histological and Histomorphometric Analysis

After 3 and 6 months, the implants together with the surrounding tissues were removed and fixed in 10% neutral buffered formalin and decalcified. The decalcification method utilized Osteomoll Merck KbaA (Darmstadt, Germany) that contained HCl (10%) and CH_2_O (4%), immersing samples for 17 days, and the solution was renewed every 24 h. Subsequently, all the samples were paraffin embedded, sectioned at 5 μm, and stained using hematoxylin-eosin (H-E, red stain) and Masson’s trichrome (MT) stain. The entire circumference of each section (containing bone, scaffold particles, and connective tissue) was traced manually to create an individual region of interest (ROI).

Histomorphometric evaluations consisted of taking measurements of the area of material in relation to the total measurement area. These were carried out using Image J software (developed by the National Institute of Health (NIH), Bethesda, MD, USA). Examinations were performed under a Nikon Elipse 80i microscope (Teknooptik AB, Huddinge, Sweden), equipped with an Easy Image 2000 system (Teknooptik AB, Huddinge, Sweden). Images were generated using a Leica Z6 APO microscope connected to a Leica DC 500 (Barcelona, Spain) digital camera. After calibrating the system and digitalizing images, interactive measurements of the areas of interest were obtained with the Leica QWin V3 image analysis software (Barcelona, Spain). The histomorphometric analysis produced one BIC measurement, measured as the percentage of the circumference, and length of the cylinder that came into contact with new bone. In the same way, the cortical bone defect in the control group was also evaluated.

### 4.5. Scanning Electron Microscopy Study

To assess the continuing effect of the implant in the medullary cavity surrounded by hematopoietic bone marrow from an ultrastructural point of view, cross-sections of the non-decalcified tissues were also examined for the ultrastructural study in SEM-EDS. Therefore, some sections (1–2 mm) in the implantation area were fixed by immersion in 3% glutaraldehyde after being soaked in buffer solution for 4 h, postfixed in 1% osmium tetroxide for 1 h at 4 °C, and washed in 0.1 M cacodilate solution and dehydrated in graded ethanol solutions series, and embedded in hydroxyethyl methacrylate resin. They were then polished with 1-μm diamond pastes for the SEM-EDS analyses. Back-scattered SEM imaging was employed to highlight the contrasts among the resin, bone and the biomaterial. 

### 4.6. Statistical Analysis

A statistical analysis was performed with the PASW Statistics v.20.0.0 software (SPSS Inc., Armonk, NY, USA). Sample size was pre-calculated using the statistical method provided by the software. Values were recorded as means ± standard deviation and medians. The pre-statistical analysis of sample distribution was performed to evaluate normality. Kolmogorov and Smirnov’s test values were normal. A non-parametric Friedman Test for the related samples was applied to the comparison of the medians and to quantify any relationships between differences (*p* < 0.05). 

## 5. Conclusions

The biocompatibility of this novel porous calcium silicophosphate, developed by the polymer replication method, was high and caused no local or systemic immune inflammatory response, and no fibrosis was developed between the ceramic and bone after its intramedullary implantation into a rabbit tibia. 

The results indicate that this material provides an optimal microenvironment for the osteogenic differentiation of the undifferentiated osteoblastic precursor cells contained in hematopoietic bone marrow. Their suitable interconnected network porous structure facilitates colonization by new bone tissue and bone marrow. Presence of the vascular capillaries that surround the material promotes this process by providing all the necessary nutrients. 

This confirms that the porous calcium silicophosphate obtained in this work favors and supports the integration of bone by combining osteoconductive behavior with the enhanced bone tissue in-growth of the open-pore structure. The dynamic biodegradation of the ceramic with time was also documented. Densities were reduced throughout the study as a result of the simultaneous phagocytic activity of macrophages. 

Finally, we therefore conclude that this novel porous calcium silicophosphate ceramic scaffold implanted into the medullary cavity is biocompatible, bioresorbable, osteoconductive and has osteogenic potential. Hence, it can be considered a potential substitute of bone tissue, and is suitable for clinical applications in filling bone defects and being used as a scaffold or matrix for bone tissue engineering.

## Figures and Tables

**Table 1:** Relevant histological findings observed by light microscopy at three and six months.

Presence, Location and Quality New Bone Formed		Fibrous Connective Tissue Formation		Inflammatory Cells Infiltration		Foci of Necrosis		Presence of Material Debris and Granulomas		Bone Marrow Changes	
3 m	6 m	3 m	6 m	3 m	6 m	3 m	6 m	3 m	6 m	3 m	6 m
- Periphery - Close contact - Osteoid	- Periphery - Inside - Osteoid - Mature	NO	NO	NO	NO	NO	NO	NO	NO	NO	NO

**Table 2:** Histomorphometric analysis to evaluate the BIC for the scaffold. Non parametric Friedman test. Significant differences (* *p* < 0.05). Control samples *n* = 4. Mean ± SD (Median).

	Implant Scaffold			Control	
%	3 Months	6 Moths	*p* Values *	3 Months	6 Moths
BIC	58.34 ± 0.12 (58.34)	67.30 ± 1.41 (67.30) *	0.040	0.00 ± 0.0	0.00 ± 0.0
New Bone	51.66 ± 0.75 (51.66)	62.18 ± 2.28 (62.18) *	0.012	0.00 ± 0.0	0.00 ± 0.0
Residual	30.05 ± 1.13 (30.05) *	24.95 ± 0.99 (24.95)	0.013	0.00 ± 0.0	0.00 ± 0.0
Defect Closure	68.53 ± 1.62 (68.53)	80.08 ± 1.96 (80.08) *	0.025	24.84 ± 1.69 (24.85)	67.63 ± 1.70 (67.64)
Connective Tissue	18.29 ± 1.28 (18.29)	12.87 ± 1.45 (12.87)	0.041	0.00 ± 0.0	0.00 ± 0.0

**Table 3:** EDS analysis with the Si (wt %) and Ca/P molar in the new bone region; the interface and the residual biomaterial after three and six months of implantation. Mean ± SD (Median).

	3 Months		6 Months	
	Si (wt %)	Ca/P	Si (wt %)	Ca/P
Residual Scaffold	7.46 ± 1.06 (7.46)	2.62	6.89 ± 1.04 (6.89)	2.68
Interphase	0.26 ± 0.24 (0.26)	2.20	0.12 ± 0.46 (0.12)	2.36
New bone	0.07 ± 1.03 (0.07)	1.97	0.04 ± 1.11 (0.04)	1.89
